# Surgical Management of Solitary Extrapulmonary Lymphangioleiomyomatosis in the Mesentery: A Case Report

**DOI:** 10.7759/cureus.69042

**Published:** 2024-09-09

**Authors:** Jack Menzie, Chih C Kuan, Travis Ackermann, Yeng Kwang Tay

**Affiliations:** 1 Department of General Surgery, Monash Health, Melbourne, AUS; 2 Department of Pathology, Monash Health, Melbourne, AUS; 3 Department of Upper Gastrointestinal and Hepatobiliary Surgery, Monash Health, Melbourne, AUS; 4 Department of Colorectal Surgery, Monash Health, Melbourne, AUS

**Keywords:** abdominal cystic mass, abdominal masses, anatomical pathology, chronic abdominal pain, diagnostic laparoscopy, exploratory laparotomy, extra-pulmonary manifestations, lymphangioleiomyoma, pulmonary lymphangioleiomyoma, unexplained abdominal pain

## Abstract

Pulmonary lymphangioleiomyomatosis (LAM) is a rare condition characterised by infiltration of the lungs with abnormal smooth muscles and cystic lesions. A rarer form of the condition is extrapulmonary LAM (E-LAM) where the same cystic lesions are found in various organs throughout the body resulting in a variety of symptoms. Given the rarity of E-LAM and the difficulty in diagnosing it, there is little evidence to guide its management both surgically and medically. We describe a case of a 22-year-old female with a nine-month history of abdominal pain found to have a large mesenteric mass during laparoscopy for suspected ovarian torsion. She underwent a laparotomy to exteriorise the mass and dissect it off the mesentery. The mass was removed without compromise to the bowel or mesentery. The patient recovered well without symptoms and was discharged without complications. Histopathology of the mass revealed it to be E-LAM. This case is the first to our knowledge that demonstrates the successful removal of a solitary E-LAM from the mesentery with minimal adverse outcomes and symptomatic relief.

## Introduction

Pulmonary lymphangioleiomyomatosis (LAM) is a rare disorder of the respiratory system that is characterized by infiltration of the lungs with abnormal smooth muscle cells and cystic lesions [1]. First described by Von Stössel in the 1930s as muscular cirrhosis of the lungs, LAM was discovered in an autopsy of a 43-year-old woman who showed diffuse cystic changes, lymphadenopathy, and microscopic proliferation of smooth muscle cells in her lungs [2]. LAM has been reported as having a predominance in women, it can occur sporadically as well as in individuals genetically predisposed to the tuberous sclerosis gene complex (TSC), a condition characterized by hamartoma-like tumour growths [3-5].

Patients presenting with LAM may show symptoms of progressive dyspnoea, cough, haemoptysis, recurrent pneumothorax, chylothorax, progressive respiratory failure, and characteristic features of numerous thin-walled cysts throughout the lungs on imaging [4,6-8]. Prevalence of the disease is rare with the sporadic condition seen in three to five women per million in the general population, whereas those with TSC show a predominance of up to 50% for women and 10% for men [8-10]. Whilst normally considered to be a respiratory condition, there are cases of extrapulmonary LAM (E-LAM). E-LAM is very rare, it can occur prior to the diagnosis or concurrently with LAM and has primarily been found in the mediastinum, retroperitoneum, lymph nodes, pelvis, uterus, and kidney hilus [8-10].

E-LAM is difficult to diagnose as it can present asymptomatically, it also grows in atypical locations and symptoms such as nausea, bloating, abdominal distention and abdominal pain are often vague making investigations delayed [6-8]. Due to the rarity of this condition and the lack of comprehensive studies, diagnosis of this condition is often not made until after surgery [9].

This case study is, to the best of our knowledge, the only case of E-LAM presenting as a solitary large mass adhered to the mesentery of the jejunum that subsequently underwent successful removal without small bowel compromise.

## Case presentation

**Figure 1:**
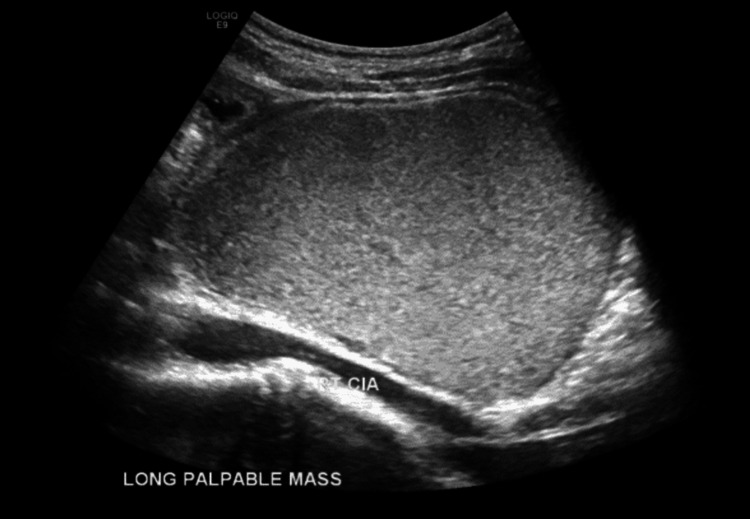
Ultrasound image of a complex mesenteric or ovarian cyst or a solid lesion such as desmoid tumor

**Figure 2:**
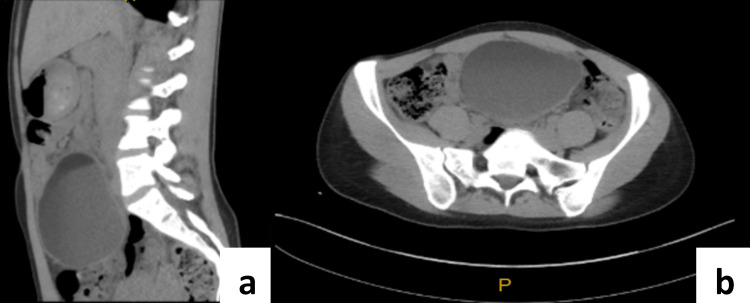
CT images (a: sagittal, b: axial) showing a large central abdominal mass that appears cystic with a fatty component anterosuperiorly and shows no enhancement, most likely a large left ovarian dermoid cyst.

A 22-year-old Caucasian female was admitted to the hospital and underwent an emergency laparoscopy for a suspected ovarian torsion. Her presentation was preceded by a nine-month history of intermittent episodes of non-specific abdominal pain, reflux, nausea, and vomiting. She denied any respiratory symptoms including cough, dyspnoea, or sputum production. Her medical and family history was negative for any significant respiratory or abdominal conditions. She denied having any urinary or bowel symptoms and at the time was not sexually active nor had ever been pregnant. On multiple presentations across seven months, biochemistry, urinalysis, and chest X-rays were normal. At approximately the seventh month of symptoms, she was found to have a palpable abdominal mass, so ultrasound (Figure 1) and computed tomography (CT) (Figure 2) investigations were organised. These revealed a large 10cm cystic lesion raising suspicion of an ovarian dermoid cyst and a gynaecology clinic appointment was arranged.

On the day of admission, the patient developed severe abdominal pain and underwent an additional ultrasound which was concerning for ovarian torsion. She promptly presented to the emergency department and was taken to the theatre for an emergency laparoscopy with the gynaecology team.

**Figure 3:**
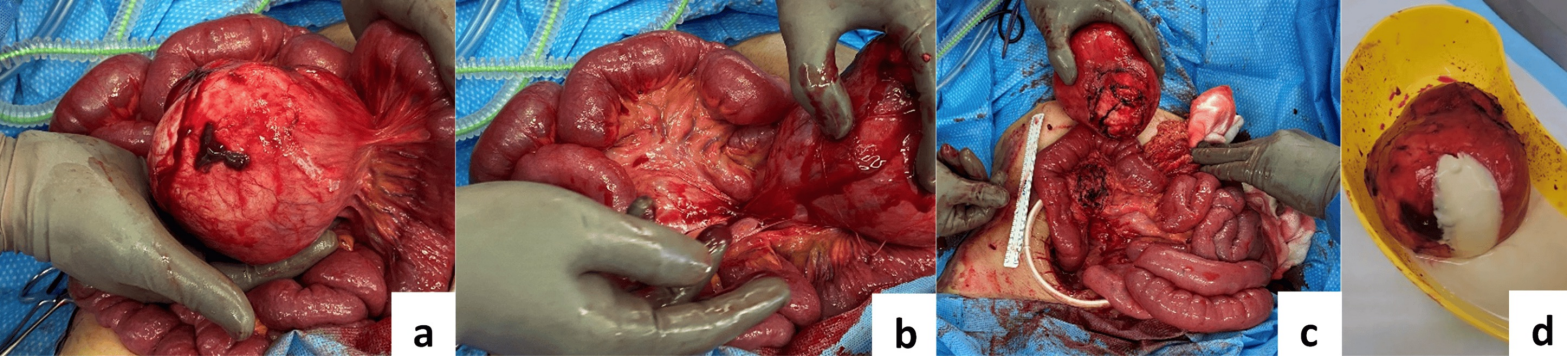
Intraoperative photos of mesenteric mass pre- and post-excision. Pre-excision of mesenteric mass (a, b). Post-excision of mass, showing healthy mesentery and small bowel (c). Incision into mass to check its contents, showing a yellow milky fluid (d).

Intraoperative findings found her to have a large (12 x 12cm) mass (Figures 3a, 3b) adhered to the mesentery of the small bowel, in addition to ischaemic appearing small bowel at the site of the mass. General Surgery was referred to take over the case and performed an emergency laparoscopic to open resection of the mesenteric mass. A midline incision was made inferior to the umbilicus to exteriorise the mass. Omentum adherent to the mass was dissected off with diathermy and ligature. Small bowel mesenteric torsion was identified as causing venous ischaemia, this was detorted and the ischaemia instantly normalised. The mesenteric mass was identified to have adhered to one side of the proximal jejunal mesentery approximately 20cm from the duodenojejunal (DJ) flexure. The mass was gently peeled off the mesentery preserving blood supply to the mesentery and jejunum (Figure 3c). Haemostasis was achieved with 3.0 PDS sutures (Ethicon, Inc., Somerville, USA) and TISSEEL (Baxter International Inc., Deerfield, USA). The abdomen was then washed with normal saline until clear and bilateral transverse abdominus plane (TAP) blocks were placed. Further inspection showed no peritoneal or small bowel lesions from DJ flexure to the ileocaecal valve. The liver was palpated, and nil abnormalities were detected. Fascia was closed with 1 PDS and skin with 3.0 monocryl. After removal, the mass was incised (Figure 3d) to inspect its contents. It contained a thick yellow milky fluid and no solid components. Swabs for microscopy culture and sensitivity (MCS) were taken including fluid for triglyceride analysis. Post-operatively, the patient went to the ward where she remained for four days without complications. She was commenced on a normal diet, her pain was well controlled, and her bowels opened on day 4. She was then discharged home and outpatient follow-up was arranged.

**Figure 4:**
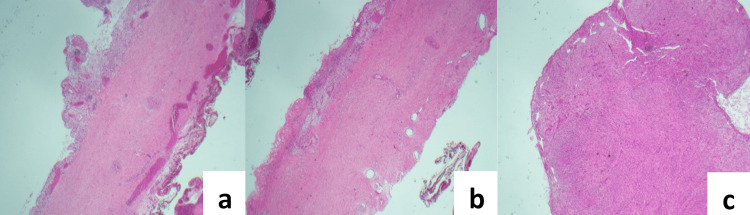
(a, b, c) Low power view of the lesion showing thickened muscular cyst wall H&E stain (hematoxylin and eosin), at 4x magnification.

**Figure 5:**
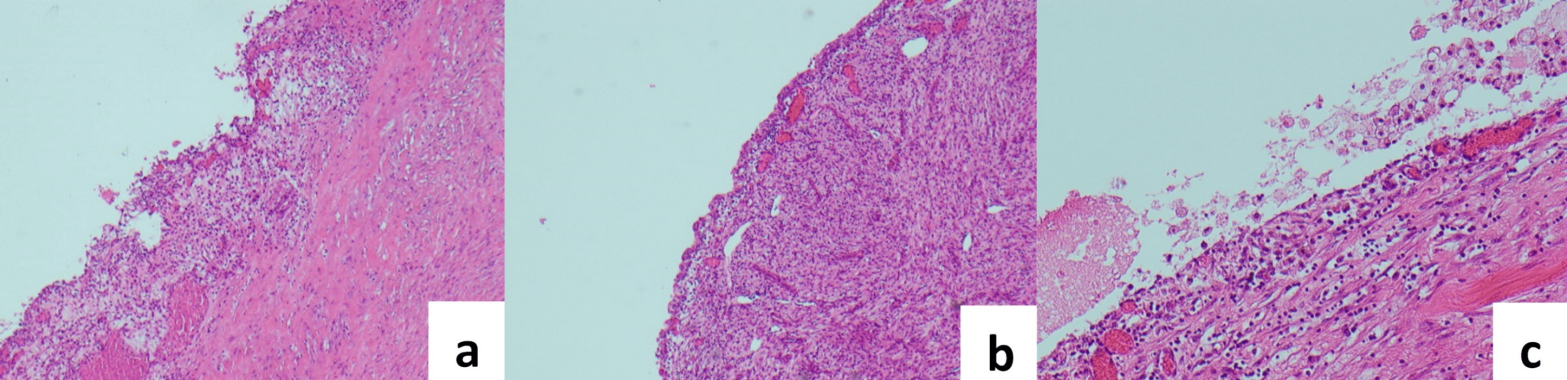
(a, b, c) Sections showing attenuated cyst lining with a variably flattened to cuboidal epithelial layer with associated inflammation rich in foamy macrophages. (a, b) H&E (haematoxylin and eosin) stain, at 10x magnification; (c) H&E stain, at 20x magnification.

**Figure 6:**
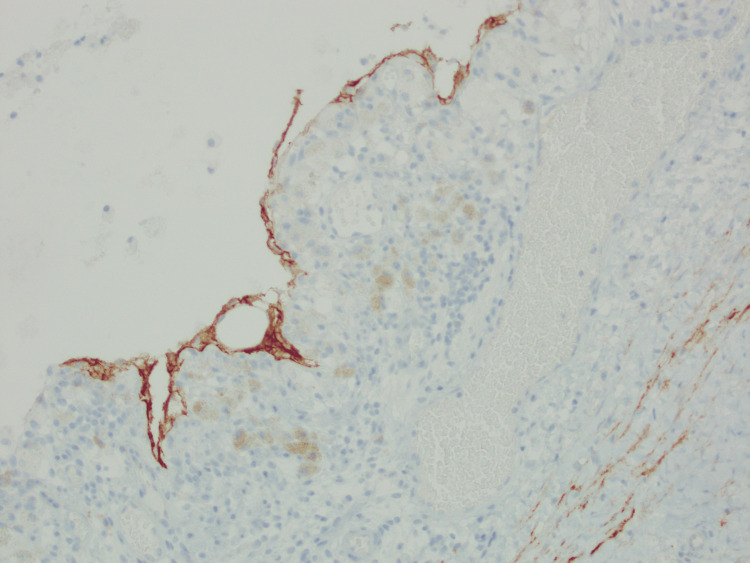
The cyst lining stains positive, confirming the presence of lymphatic differentiation in the epithelial lining. D2-40 stain, at 10x magnification.

**Figure 7:**
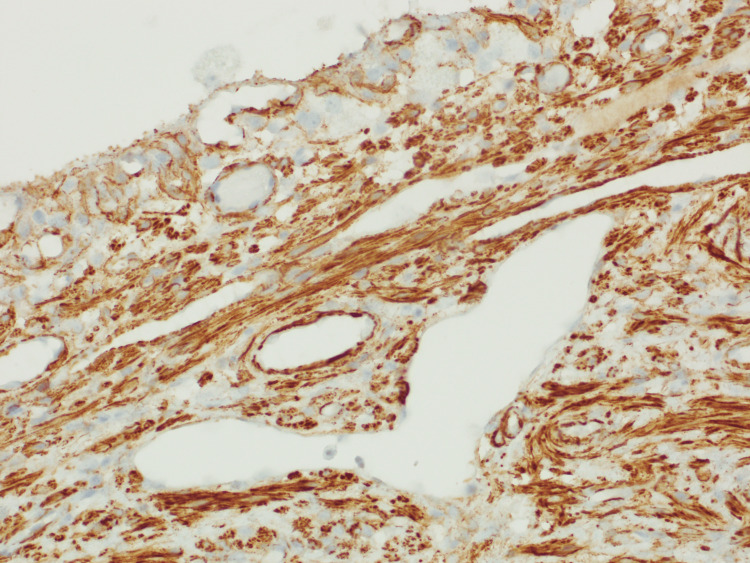
Stroma cells show diffuse positive staining within the cyst wall, consistent with smooth muscle differentiation. H-caldesmon stain, at 20x magnification.

Fluid from the cystic mass showed no growth on MCS, the fluid specimen was deemed unsuitable for analysis of triglycerides and so nil testing was performed. Macroscopic histopathology revealed a 10mm thick wall (Figures 4a-4c) with the inner surface being irregular and roughened, covered in a milky material. Nil discrete lesions or papillary excrescences were identified. Microscopically, there was an intact flattened epithelial lining (Figures 5a-5c), with a D2-40 stain (Figure 6) highlighting lymphatic spaces within the cyst wall. The cyst wall also contained large amounts of smooth muscle tissue which showed diffuse and strong positive staining for desmin, actin, and caldesmon (Figure 7). The lesion was deemed to be completely excised with no malignancy present. Additional stains for HMB45, SOX10, and S100 were negative. Overall, the cystic lesion comprised benign proliferation of lymphatic vessels and smooth muscle cells that are most consistent with an LAM.

## Discussion

LAM is a rare condition characterized by the proliferation of abnormal smooth muscle cells and lymphatics leading to cystic lung lesions as well as other phenomena such as abdominal tumours and chylous fluid collections [1,7]. The condition occurs mainly in women, primarily of reproductive age with an average age of onset at 35-41 years old [4,7,9]. Prevalence of the condition is approximately three to five in one million women with increased prevalence in those with TSC and some differences reported amongst Western and Asian populations [7,11]. LAM presents with symptoms such as cough, haemoptysis, progressive dyspnea, recurrent pneumothorax, chylothorax, and respiratory failure leading to difficulty in diagnosis. Often misdiagnosed as asthma or chronic obstructive pulmonary disease (COPD), a formal diagnosis can incur delays for several years until after the failure of conventional treatments [1,12]. Upon diagnosis of the condition, without a lung transplant, the median survival of patients with LAM is 94% at five years, 85% at 10 years, 75% at 15 years and 64% at 20 years [13].

The exact cause of the condition is unknown, it occurs in two forms: a sporadic form and a TSC form which shows a genetic predisposition to the condition [1,9]. Whilst primarily a systemic lung condition, it can present as an extrapulmonary condition concurrently or preceding respiratory signs and symptoms [1,9]. Solitary E-LAM, however, is very rare, difficult to diagnose, and often presents as a single well-circumscribed mass [8,9]. E-LAM has been well documented in previous studies and often occurs in the posterior mediastinum, the upper retroperitoneum, or in the pelvis [8,9]. There have been few cases of E-LAM reported in other areas, with a handful of cases describing E-LAM in the mesentery and one case involving the liver [9,14-16]. In the case described by Possekel et al. [15], a 23-year-old female presented with diffuse abdominal pain, and subsequent CT imaging showed multiple intrahepatic and mesenteric cystic lesions. Additional cystic lesions were noted in the small and large bowel walls and exploratory laparotomy showed immunochemistry findings positive for smooth-muscle-like cells which were positive for smooth-muscle actin and melan-A. Due to the diffuse nature of the cystic lesions, the patient did not undergo resection and was commenced on sirolimus therapy which showed some improvement in cyst size at three months [15]. Unlike our case study, the above case did not show a solitary lesion but included cysts in additional regions not previously seen, similarly to our case the patient showed no lung disease [15]. From the literature research, and to the best of our knowledge, there have only been three previous cases of a solitary E-LAM tumour involving the mesentery of the small bowel and one case involving mesenteric lymphangiomatosis in an 11-year-old female [14,16,17].

In the case of the 11-year-old female, she was affected by lymphangiomatosis which similarly to LAM is a rare condition characterised by tumours involving the lymphatic system [18]. However, they differ by pathogenesis with lymphangiomatosis showing a characteristic proliferation, dilatation, and thickening of lymphatic vessels without the characteristic cystic lesions and thick smooth muscle walls seen in LAM [18]. Lymphangiomatosis shows the proliferation of channels lined by spindled endothelial cells with the absence of smooth muscle, whereas LAM shows a combination of smooth muscle and lymphatic proliferation with prominent cyst changes and oftentimes reactivity to monoclonal antibody HMB45 (although absent in our case). Furthermore, lymphangiomatosis shows cells that react with anti-CD31 and lack other characteristics associated with LAM such as estrogen receptors and S-100 protein [14,16,18]. The treatment of E-LAM and LAM has often been focused on anti-estrogen therapy and more recently immunosuppressive treatment with the agent sirolimus [1,15,19]. Whilst this has shown some benefit for the systemic disease, our case involves a solitary mass in the mesentery without lung disease causing abdominal symptoms and reduced quality of life [1,19].

Despite a differing diagnosis, the surgical management in the case of the 11-year-old has previously been compared to the management for E-LAM [14,17]. A laparotomy was performed which revealed a multi-nodular (8 x 6 x 7cm) mass at the root of the mesentery encircled by the superior mesenteric vessels resulting in incomplete resection of the tumour and subsequent conservative management [17]. According to the study at 10 years of follow-up, the tumour had remained stable and the patient was asymptomatic [17].

Matsui et al. [14] compared 22 cases of E-LAM, two cases presented with singular mesenteric masses measuring 8cm in a 43-year-old female and 15cm in a 51-year-old female. Both cases involved resection of the masses using a laparotomy as the approach, however, it is unclear what the intraoperative steps were and if any bowel resection or complications occurred [14,16]. Both cases had pulmonary LAM one of which was diagnosed after resection, additionally, one patient had further lesions found in the form of angiolipomas [14].

Only one case in the literature has described an E-LAM mass involving the mesentery that did not include other manifestations of the disease or lung involvement [16]. The case report from South Korea by Jun et al. [16] involved a 47-year-old woman who presented with a seven-year history of palpable abdominal mass and nil significant medical history [16]. A CT scan revealed a well-marginated mass with soft tissue attenuation and a multifocal enhanced area, located at the mesenteric area and abutted to the bowel loop [16]. The case further describes the resection of the mass using a laparoscopic approach and the mass measuring 17 x 12 x 10cm [16]. Whilst intraoperative steps are not discussed in the case, photos of the mass were included in the report which appear to show resection of the mass described including the mesentery and the adjacent small bowel.

It is difficult to compare surgical approaches in the literature as previous case reports have focused on the immunochemistry of the disease rather than the anatomy or surgical approach involved. In comparison to our case, we describe a large mesenteric mass adhered to the mesentery of the jejunum that measured 12 (L) x 12 (W) cm and had a circumference of 30cm. Our surgical approach involved laparoscopy with conversion to an inferior midline laparotomy for better access to the mass, avoiding damage to the surrounding intraabdominal structures while ensuring the mass was removed intact. By using this approach, we were able to carefully dissect the mass off the wall of the mesentery leaving the mesentery the mass was adhered to and the adjacent small bowel intact. Additionally, our patient had an uncomplicated admission and complete resolution of her symptoms without requiring further medical management such as estrogen or sirolimus therapies.

To the best of our knowledge, ours is the first case of a large mesenteric E-LAM mass resected en bloc without the need to resect the mesentery or adjacent small bowel, thereby potentially reducing post-operative complications. Given the rarity of this condition and the few cases involving the mesentery of the small bowel in the literature, there is insufficient evidence to determine the most appropriate approach to treating solitary E-LAM. Additionally, previous cases of mesenteric E-LAM have involved the lungs and other organs suggesting the need for medical therapy [1,14,19]. In one case without systemic involvement, the patient had a 10-month disease-free period from the condition and studies suggest that diagnosis of LAM may occur up to two years post-solitary findings in the abdomen [7,14,16]. Due to the lack of surgical cases involving E-LAM and the literature showing little evidence for conservative versus active treatment for E-LAM, physicians have no pathway to suggest what the most appropriate management for this condition is. What is clear from our case is that in the case of solitary E-LAM masses involving the mesentery, a surgical approach can provide one option for surgeons to treat patients who are symptomatic at least initially. However, further evidence in the form of case reports on surgical approaches for these solitary lesions is needed as well as a further understanding of the disease at large.

## Conclusions

Solitary E-LAM tumours are very rare and can present with a variety of symptoms and locations, making diagnosis difficult and therefore treatment challenging for physicians. Our case suggests that if a solitary lesion is adhered to the mesentery, it can be successfully resected with minimal adverse outcomes and symptomatic relief for the patient. However, given the literature on LAM, careful monitoring and follow-up are needed to assess if there is a progression of the disease. Additionally, given that E-LAM tumours can present in a variety of locations and be of differing sizes, further surgical cases are required in the literature to provide surgeons with comprehensive evidence on how to best approach these cases in the future.
